# [2,2′-(1,1′-Binaphthyl-2,2′-diyldiimino)­diethanol-κ^3^ *N*,*N*′,*O*]dichloridocopper(II)

**DOI:** 10.1107/S1600536811049828

**Published:** 2011-11-25

**Authors:** Wan-Yun Huang, Dong-Cheng Liu, Han-Chang Wei, Fu-Pei Liang

**Affiliations:** a College of Chemistry and Chemical Engineering, Guangxi Normal University, Yucai Road 15, Guilin 541004, People’s Republic of China

## Abstract

In the title complex, [CuCl_2_(C_24_H_24_N_2_O_2_)], the Cu^II^ cation is *N*,*N*′,*O*-chelated by a 2,2′-(1,1′-binaphthyl-2,2′-diyldiimino)­diethanol ligand and coordinated by two chloride anions in a distorted square-pyramidal geometry. In the diethanol ligand, the two naphthalene ring systems are twisted with respect to each other at a dihedral angle of 68.30 (9)°. The uncoord­inated hy­droxy group links with a coordinated chloride anion *via* an intra­molecular O—H⋯Cl hydrogen bond. Inter­molecular N—H⋯O and N—H⋯Cl hydrogen bonds occur in the crystal structure.

## Related literature

For background to metal complexes containing *N*-substituted diethano­lamine ligands, see: Saalfrank *et al.* (2008 ▶); Ferguson *et al.* (2011 ▶); Alley *et al.* (2008 ▶). For the synthesis of the ligand, see: Yan *et al.* (2008 ▶). For related structures, see: Thob *et al.* (2010 ▶); Telfer *et al.* (2004 ▶).
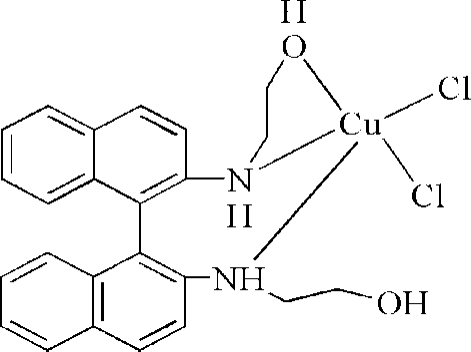


## Experimental

### 

#### Crystal data

[CuCl_2_(C_24_H_24_N_2_O_2_)]
*M* *_r_* = 506.89
Triclinic, 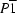

*a* = 7.4816 (8) Å
*b* = 10.4211 (11) Å
*c* = 15.2116 (16) Å
α = 94.130 (2)°
β = 103.633 (2)°
γ = 106.912 (2)°
*V* = 1090.1 (2) Å^3^
*Z* = 2
Mo *K*α radiation
μ = 1.27 mm^−1^
*T* = 185 K
0.31 × 0.17 × 0.10 mm

#### Data collection

Bruker SMART 1000 CCD area-detector diffractometer
Absorption correction: multi-scan (*SADABS*; Bruker, 2001 ▶) *T*_min_ = 0.694, *T*_max_ = 0.883
5452 measured reflections
3758 independent reflections
3363 reflections with *I* > 2σ(*I*)
*R*_int_ = 0.012

#### Refinement

*R*[*F* ^2^ > 2σ(*F* ^2^)] = 0.037
*wR*(*F* ^2^) = 0.107
*S* = 1.04
3758 reflections
280 parameters
H-atom parameters constrained
Δρ_max_ = 0.53 e Å^−3^
Δρ_min_ = −0.40 e Å^−3^

### 

Data collection: *SMART* (Bruker, 2007 ▶); cell refinement: *SAINT* (Bruker, 2007 ▶); data reduction: *SAINT*; program(s) used to solve structure: *SHELXTL* (Sheldrick, 2008 ▶); program(s) used to refine structure: *SHELXTL*; molecular graphics: *SHELXTL*; software used to prepare material for publication: *SHELXTL*.

## Supplementary Material

Crystal structure: contains datablock(s) global, I. DOI: 10.1107/S1600536811049828/xu5393sup1.cif

Structure factors: contains datablock(s) I. DOI: 10.1107/S1600536811049828/xu5393Isup2.hkl

Additional supplementary materials:  crystallographic information; 3D view; checkCIF report

## Figures and Tables

**Table 1:** Selected bond lengths (Å)

Cu1—N1	2.052 (2)
Cu1—N2	2.106 (2)
Cu1—O2	1.965 (2)
Cu1—Cl1	2.6190 (7)
Cu1—Cl2	2.2272 (8)

**Table 2:** Hydrogen-bond geometry (Å, °)

*D*—H⋯*A*	*D*—H	H⋯*A*	*D*⋯*A*	*D*—H⋯*A*
O1—H1*A*⋯Cl1	0.84	2.41	3.039 (2)	132
O2—H2*B*⋯Cl1^i^	0.84	2.33	2.996 (2)	137
N2—H2*A*⋯O1^ii^	0.93	2.00	2.889 (3)	158

Symmetry codes: (i) 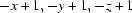; (ii) 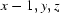.

**Fig. 1.**
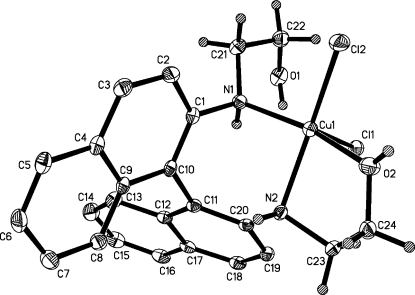
The molecular structure of the title compound with the atom-numbering scheme and 30% displacement ellipsoids. H atoms of the Aryl group are omitted for clarity.

**Fig. 2.**
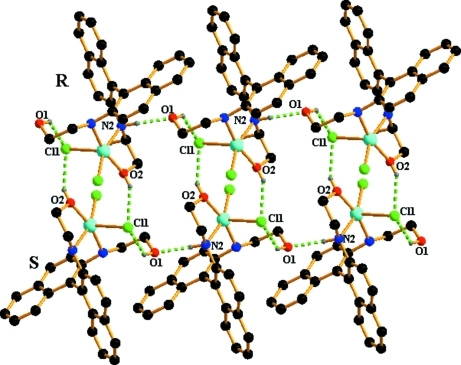
Two adjacent polymeric H-bonded chains of opposite chirality in the crystal structure, viewed along the a direction. H atoms of the Aryl group are omitted for clarity. Hydrogen bonds are shown as dashed lines. [Symmetry codes: (i) x - 1, y, z; (ii) -x + 1, -y + 1, -z + 1].

## supplementary crystallographic information

### Comment

N-substituted diethanolamine ligands have been proven to be fruitful in the construction of fascinating functional metal complexes (Saalfrank *et al.*, 2008; Ferguson *et al.*, 2011; Alley *et al.*, 2008). But the ligand which contains two or more N-substituted diethanolamine has received much less attention. we designed and synthesized successfully a racemic ligand of N, *N*'-Bis-(2-hydroxy-ethyl)-(1,1'-Binaphthyl-2,2'-diamine) (BHEBA), herein we report the crystal structure of a Cu^II^ complex about it. The single-crystal X-ray structural analysis reveals that asymmetric unit contains one five-coordinated Cu^II^ ion, a new ligand *N*,*N*'-(2-hydroxy-ethyl)-(1,1'-binaphthyl-2,2'-diamine) (HEBA), namely partly decomposed of the parent ligand and two Cl^-^ ions as shown in Fig. 1. Due to the Jahn-Teller effect, the distance of the Cu—Cl(1) bond is elongated to 2.6188 (Å), which is consistent with the reported copper complexes (Telfer *et al.*, 2004). Two adjacent polymeric H-bonded chains of opposite chirality (Thob *et al.*, 2010) by the hydrogen bond N(2)—H(2 A)···O(1) and O(1)—H (1 A)···Cl(1) extend along *a* direction, and these chains are interconnected by the repeating weak O(2)—H(2B)···Cl(1) (symmetry code: -*x* + 1, -*y* + 1, -*z* + 1) hydrogen bonds (Fig. 2.), which further stabilize the structure. The corresponding lengths and angles of hydrogen bonds are listed in Table 1.

### Experimental

The target ligand of racemic N,N'-Bis-(2-hydroxy-ethyl)-(1,1'-Binaphthyl-2,2'- diamine) (BHEBA) were synthesized by the reported procedure (Yan *et al.*, 2008) in 55% yield using racemic 1,1'-binaphthyl-2,2'-diamine as materials.

CuCl_2_ (25.5 mg, 0.15 mmol), NMe_4_OH 18.1 mg (0.10 mmol), and BHEBA (46.1 mg, 0.10 mmol) were mixed in a CH_3_OH /^i^PrOH (10 ml, *v*/*v* 3:2) solution with vigorous stirring for 10 h. The resulting solution was filtered and left to stand at room temperature. Brown block crystals suitable for X-ray analysis were obtained in 30% yield by slow evaporation of the solvent over a period of two week. Analysis, calculated for C_24_H_24_N_2_O_2_Cl_2_Cu: C 56.86, H 4.77, N 5.53%; found: C 56.45, H 4.43, N 5.62%.

### Refinement

H atoms were placed in geometrically calculated positions and refined as riding atoms, with C—H = 0.95 (aromatic) or 0.99 Å (CH2) and O—H = 0.84 and N—H = 0.93 Å, U_iso_(H) = 1.2U_eq_(C,N) and 1.5U_eq_(O).

### Crystal data

**Table:**

[CuCl_2_(C_24_H_24_N_2_O_2_)]	*Z* = 2
*M**_r_* = 506.89	*F*(000) = 522
Triclinic, *P*1	*D*_x_ = 1.544 Mg m^−^^3^
Hall symbol: -P 1	Mo *K*α radiation, λ = 0.71073 Å
*a* = 7.4816 (8) Å	Cell parameters from 3367 reflections
*b* = 10.4211 (11) Å	θ = 2.7–26.0°
*c* = 15.2116 (16) Å	µ = 1.27 mm^−^^1^
α = 94.130 (2)°	*T* = 185 K
β = 103.633 (2)°	Block, brown
γ = 106.912 (2)°	0.31 × 0.17 × 0.10 mm
*V* = 1090.1 (2) Å^3^	

### Data collection

**Table:**

Bruker SMART 1000 CCD area-detector diffractometer	3758 independent reflections
Radiation source: fine-focus sealed tube	3363 reflections with *I* > 2σ(*I*)
graphite	*R*_int_ = 0.012
φ and ω scans	θ_max_ = 25.0°, θ_min_ = 2.1°
Absorption correction: multi-scan (*SADABS*; Bruker, 2001)	*h* = −8→8
*T*_min_ = 0.694, *T*_max_ = 0.883	*k* = −12→12
5452 measured reflections	*l* = −18→11

### Refinement

**Table:**

Refinement on *F*^2^	Primary atom site location: structure-invariant direct methods
Least-squares matrix: full	Secondary atom site location: difference Fourier map
*R*[*F*^2^ > 2σ(*F*^2^)] = 0.037	Hydrogen site location: inferred from neighbouring sites
*wR*(*F*^2^) = 0.107	H-atom parameters constrained
*S* = 1.04	*w* = 1/[σ^2^(*F*_o_^2^) + (0.070*P*)^2^ + 0.7465*P*] where *P* = (*F*_o_^2^ + 2*F*_c_^2^)/3
3758 reflections	(Δ/σ)_max_ = 0.001
280 parameters	Δρ_max_ = 0.53 e Å^−^^3^
0 restraints	Δρ_min_ = −0.40 e Å^−^^3^

### Special details

**Table:**

Experimental. The elemental analysis, Ms, IR and ^1^H NMR, ^13^C NMR of the ligand are all in good agreement with the assumed structure. Analysis calculated (%) for C_28_H_32_N_2_O_4_: C, 73.02; H, 7.00; N, 6.08; Found: C, 73.53; H, 7.62; N, 5.76. IR (KBr, cm^-1^): 3369(*s*), 3055(w), 2936(*m*), 1617(*s*), 1594(*s*), 1504(*s*), 1468(*m*), 1424(*m*), 1358(*s*), 1199(w), 1147(*m*), 1046(*s*), 817(*s*), 750(*s*). ^1^H NMR (DMSO, 500 MHz) σ: 7.93–6.85 (m, 12H, Ar*H*), 4.26 (m, 4H, CH_2_O*H*), 3.09 (*M*, 8H, C*H*_2_OH), 2.96 (*M*, 8H, NC*H*_2_CH_2_OH); ^13^C NMR (DMSO, 125.77 MHz) σ: 148.24, 134.54, 130.03, 128.80, 128.37, 126.51, 126.45, 125.88, 124.09, 122.93, 59.86, 55.67. ESI-Ms: M—H^-^ peak at *m/z* 458.53.
Geometry. All e.s.d.'s (except the e.s.d. in the dihedral angle between two l.s. planes) are estimated using the full covariance matrix. The cell e.s.d.'s are taken into account individually in the estimation of e.s.d.'s in distances, angles and torsion angles; correlations between e.s.d.'s in cell parameters are only used when they are defined by crystal symmetry. An approximate (isotropic) treatment of cell e.s.d.'s is used for estimating e.s.d.'s involving l.s. planes.
Refinement. Refinement of *F*^2^ against ALL reflections. The weighted *R*-factor *wR* and goodness of fit *S* are based on *F*^2^, conventional *R*-factors *R* are based on *F*, with *F* set to zero for negative *F*^2^. The threshold expression of *F*^2^ > σ(*F*^2^) is used only for calculating *R*-factors(gt) *etc*. and is not relevant to the choice of reflections for refinement. *R*-factors based on *F*^2^ are statistically about twice as large as those based on *F*, and *R*-factors based on ALL data will be even larger.

### Fractional atomic coordinates and isotropic or equivalent isotropic displacement parameters (Å^2^)

**Table:**

	*x*	*y*	*z*	*U*_iso_*/*U*_eq_	
Cu1	0.45715 (5)	0.32140 (3)	0.60514 (2)	0.02338 (13)	
Cl1	0.78869 (9)	0.51803 (6)	0.65550 (5)	0.02669 (18)	
Cl2	0.43545 (12)	0.20440 (8)	0.47249 (5)	0.0382 (2)	
C1	0.3922 (4)	0.1142 (3)	0.71308 (18)	0.0228 (6)	
C2	0.2759 (4)	−0.0126 (3)	0.6591 (2)	0.0299 (6)	
H2	0.3104	−0.0450	0.6076	0.036*	
C3	0.1151 (4)	−0.0876 (3)	0.6810 (2)	0.0307 (6)	
H3	0.0398	−0.1732	0.6452	0.037*	
C4	0.0582 (4)	−0.0404 (3)	0.75604 (19)	0.0248 (6)	
C5	−0.1103 (4)	−0.1166 (3)	0.7790 (2)	0.0312 (6)	
H5	−0.1858	−0.2029	0.7442	0.037*	
C6	−0.1658 (4)	−0.0684 (3)	0.8502 (2)	0.0355 (7)	
H6	−0.2783	−0.1210	0.8653	0.043*	
C7	−0.0546 (4)	0.0606 (3)	0.9013 (2)	0.0348 (7)	
H7	−0.0944	0.0953	0.9503	0.042*	
C8	0.1083 (4)	0.1355 (3)	0.8814 (2)	0.0277 (6)	
H8	0.1809	0.2218	0.9168	0.033*	
C9	0.1722 (4)	0.0877 (3)	0.80891 (18)	0.0221 (5)	
C10	0.3443 (4)	0.1644 (3)	0.78723 (17)	0.0205 (5)	
C11	0.4725 (4)	0.2994 (3)	0.84286 (18)	0.0210 (5)	
C12	0.5856 (4)	0.3070 (3)	0.93434 (18)	0.0222 (6)	
C13	0.5791 (4)	0.1920 (3)	0.9794 (2)	0.0310 (6)	
H13	0.4958	0.1052	0.9483	0.037*	
C14	0.6904 (5)	0.2038 (3)	1.0668 (2)	0.0363 (7)	
H14	0.6835	0.1251	1.0954	0.044*	
C15	0.8152 (4)	0.3313 (3)	1.1149 (2)	0.0351 (7)	
H15	0.8917	0.3385	1.1756	0.042*	
C16	0.8251 (4)	0.4438 (3)	1.0737 (2)	0.0313 (7)	
H16	0.9092	0.5296	1.1064	0.038*	
C17	0.7126 (4)	0.4358 (3)	0.98322 (18)	0.0248 (6)	
C18	0.7260 (4)	0.5509 (3)	0.9390 (2)	0.0277 (6)	
H18	0.8135	0.6363	0.9705	0.033*	
C19	0.6176 (4)	0.5433 (3)	0.85272 (19)	0.0252 (6)	
H19	0.6288	0.6230	0.8249	0.030*	
C20	0.4873 (4)	0.4167 (3)	0.80370 (18)	0.0208 (5)	
N1	0.5583 (3)	0.1950 (2)	0.68647 (15)	0.0225 (5)	
H1	0.6406	0.2511	0.7397	0.027*	
N2	0.3739 (3)	0.4081 (2)	0.71187 (15)	0.0211 (5)	
H2A	0.2494	0.3527	0.7087	0.025*	
C23	0.3526 (4)	0.5384 (3)	0.6829 (2)	0.0278 (6)	
H23A	0.4815	0.6032	0.6865	0.033*	
H23B	0.2925	0.5797	0.7239	0.033*	
C21	0.6734 (4)	0.1155 (3)	0.6551 (2)	0.0297 (6)	
H21A	0.5928	0.0530	0.5983	0.036*	
H21B	0.7091	0.0600	0.7022	0.036*	
C24	0.2268 (4)	0.5076 (3)	0.5865 (2)	0.0314 (6)	
H24A	0.0901	0.4603	0.5846	0.038*	
H24B	0.2335	0.5923	0.5600	0.038*	
C22	0.8557 (4)	0.2057 (3)	0.6372 (2)	0.0340 (7)	
H22A	0.9313	0.1489	0.6200	0.041*	
H22B	0.8197	0.2532	0.5850	0.041*	
O1	0.9733 (3)	0.3032 (2)	0.71480 (15)	0.0362 (5)	
H1A	0.9098	0.3518	0.7295	0.054*	
O2	0.3005 (4)	0.4224 (3)	0.53688 (15)	0.0452 (6)	
H2B	0.2296	0.3983	0.4829	0.068*	

### Atomic displacement parameters (Å^2^)

**Table:**

	*U*^11^	*U*^22^	*U*^33^	*U*^12^	*U*^13^	*U*^23^
Cu1	0.0258 (2)	0.0231 (2)	0.0227 (2)	0.00924 (14)	0.00696 (14)	0.00440 (13)
Cl1	0.0260 (3)	0.0260 (3)	0.0274 (4)	0.0071 (3)	0.0073 (3)	0.0045 (3)
Cl2	0.0523 (5)	0.0344 (4)	0.0266 (4)	0.0175 (3)	0.0057 (3)	−0.0017 (3)
C1	0.0228 (13)	0.0192 (12)	0.0251 (14)	0.0044 (10)	0.0060 (11)	0.0062 (10)
C2	0.0351 (16)	0.0236 (14)	0.0288 (15)	0.0053 (12)	0.0108 (13)	−0.0005 (11)
C3	0.0311 (15)	0.0207 (13)	0.0325 (16)	0.0002 (11)	0.0054 (13)	−0.0021 (12)
C4	0.0244 (14)	0.0208 (13)	0.0256 (14)	0.0045 (11)	0.0024 (11)	0.0073 (11)
C5	0.0277 (15)	0.0236 (14)	0.0384 (17)	0.0022 (11)	0.0082 (13)	0.0086 (12)
C6	0.0301 (15)	0.0339 (16)	0.0445 (18)	0.0061 (13)	0.0159 (14)	0.0160 (14)
C7	0.0376 (17)	0.0400 (17)	0.0335 (16)	0.0154 (14)	0.0168 (14)	0.0102 (13)
C8	0.0283 (14)	0.0254 (14)	0.0277 (15)	0.0066 (11)	0.0069 (12)	0.0022 (11)
C9	0.0239 (13)	0.0205 (13)	0.0211 (13)	0.0072 (10)	0.0027 (11)	0.0079 (10)
C10	0.0219 (13)	0.0181 (12)	0.0185 (13)	0.0052 (10)	0.0002 (11)	0.0060 (10)
C11	0.0193 (12)	0.0194 (12)	0.0229 (13)	0.0048 (10)	0.0058 (11)	−0.0001 (10)
C12	0.0188 (12)	0.0252 (14)	0.0219 (13)	0.0069 (10)	0.0053 (11)	−0.0007 (11)
C13	0.0319 (15)	0.0269 (14)	0.0302 (15)	0.0091 (12)	0.0016 (13)	0.0034 (12)
C14	0.0408 (18)	0.0399 (17)	0.0295 (16)	0.0174 (14)	0.0045 (14)	0.0098 (13)
C15	0.0295 (15)	0.0516 (19)	0.0222 (15)	0.0170 (14)	−0.0005 (12)	0.0002 (13)
C16	0.0227 (14)	0.0403 (17)	0.0253 (15)	0.0069 (12)	0.0035 (12)	−0.0072 (13)
C17	0.0209 (13)	0.0283 (14)	0.0239 (14)	0.0057 (11)	0.0086 (11)	−0.0028 (11)
C18	0.0243 (14)	0.0230 (13)	0.0310 (15)	0.0006 (11)	0.0100 (12)	−0.0063 (11)
C19	0.0260 (14)	0.0187 (13)	0.0316 (15)	0.0043 (11)	0.0125 (12)	0.0039 (11)
C20	0.0189 (12)	0.0202 (12)	0.0243 (14)	0.0061 (10)	0.0085 (11)	0.0002 (10)
N1	0.0224 (11)	0.0198 (11)	0.0235 (11)	0.0039 (9)	0.0071 (9)	0.0011 (9)
N2	0.0210 (11)	0.0171 (10)	0.0259 (12)	0.0056 (9)	0.0075 (9)	0.0049 (9)
C23	0.0338 (15)	0.0235 (14)	0.0329 (15)	0.0146 (12)	0.0136 (13)	0.0086 (12)
C21	0.0301 (15)	0.0256 (14)	0.0349 (16)	0.0116 (12)	0.0078 (13)	0.0052 (12)
C24	0.0334 (15)	0.0361 (16)	0.0350 (16)	0.0191 (13)	0.0152 (13)	0.0153 (13)
C22	0.0344 (16)	0.0339 (16)	0.0395 (17)	0.0158 (13)	0.0142 (14)	0.0086 (13)
O1	0.0247 (10)	0.0354 (12)	0.0463 (13)	0.0095 (9)	0.0047 (10)	0.0092 (10)
O2	0.0670 (16)	0.0607 (15)	0.0265 (11)	0.0464 (13)	0.0133 (11)	0.0121 (10)

### Geometric parameters (Å, °)

**Table:**

Cu1—N1	2.052 (2)	C14—C15	1.410 (4)
Cu1—N2	2.106 (2)	C14—H14	0.9500
Cu1—O2	1.965 (2)	C15—C16	1.360 (5)
Cu1—Cl1	2.6190 (7)	C15—H15	0.9500
Cu1—Cl2	2.2272 (8)	C16—C17	1.417 (4)
C1—C10	1.374 (4)	C16—H16	0.9500
C1—C2	1.419 (4)	C17—C18	1.407 (4)
C1—N1	1.447 (3)	C18—C19	1.353 (4)
C2—C3	1.359 (4)	C18—H18	0.9500
C2—H2	0.9500	C19—C20	1.419 (4)
C3—C4	1.413 (4)	C19—H19	0.9500
C3—H3	0.9500	C20—N2	1.435 (3)
C4—C5	1.417 (4)	N1—C21	1.486 (3)
C4—C9	1.420 (4)	N1—H1	0.9300
C5—C6	1.361 (5)	N2—C23	1.497 (3)
C5—H5	0.9500	N2—H2A	0.9300
C6—C7	1.413 (4)	C23—C24	1.499 (4)
C6—H6	0.9500	C23—H23A	0.9900
C7—C8	1.357 (4)	C23—H23B	0.9900
C7—H7	0.9500	C21—C22	1.511 (4)
C8—C9	1.414 (4)	C21—H21A	0.9900
C8—H8	0.9500	C21—H21B	0.9900
C9—C10	1.430 (4)	C24—O2	1.430 (4)
C10—C11	1.509 (3)	C24—H24A	0.9900
C11—C20	1.385 (4)	C24—H24B	0.9900
C11—C12	1.431 (4)	C22—O1	1.420 (4)
C12—C13	1.417 (4)	C22—H22A	0.9900
C12—C17	1.428 (4)	C22—H22B	0.9900
C13—C14	1.368 (4)	O1—H1A	0.8400
C13—H13	0.9500	O2—H2B	0.8400
			
O2—Cu1—N1	165.49 (10)	C15—C16—C17	121.4 (3)
O2—Cu1—N2	79.76 (9)	C15—C16—H16	119.3
N1—Cu1—N2	91.66 (8)	C17—C16—H16	119.3
O2—Cu1—Cl2	88.68 (7)	C18—C17—C16	121.8 (3)
N1—Cu1—Cl2	96.06 (7)	C18—C17—C12	118.8 (2)
N2—Cu1—Cl2	160.29 (7)	C16—C17—C12	119.3 (3)
O2—Cu1—Cl1	97.74 (8)	C19—C18—C17	121.8 (2)
N1—Cu1—Cl1	93.68 (6)	C19—C18—H18	119.1
N2—Cu1—Cl1	88.52 (6)	C17—C18—H18	119.1
Cl2—Cu1—Cl1	108.97 (3)	C18—C19—C20	120.2 (3)
C10—C1—C2	121.2 (2)	C18—C19—H19	119.9
C10—C1—N1	119.5 (2)	C20—C19—H19	119.9
C2—C1—N1	119.2 (2)	C11—C20—C19	120.4 (2)
C3—C2—C1	120.1 (3)	C11—C20—N2	119.2 (2)
C3—C2—H2	120.0	C19—C20—N2	120.3 (2)
C1—C2—H2	120.0	C1—N1—C21	114.2 (2)
C2—C3—C4	121.1 (3)	C1—N1—Cu1	105.29 (16)
C2—C3—H3	119.4	C21—N1—Cu1	120.05 (18)
C4—C3—H3	119.4	C1—N1—H1	105.4
C3—C4—C5	121.8 (3)	C21—N1—H1	105.4
C3—C4—C9	118.8 (2)	Cu1—N1—H1	105.4
C5—C4—C9	119.4 (3)	C20—N2—C23	116.3 (2)
C6—C5—C4	121.1 (3)	C20—N2—Cu1	117.44 (16)
C6—C5—H5	119.4	C23—N2—Cu1	104.06 (16)
C4—C5—H5	119.4	C20—N2—H2A	106.0
C5—C6—C7	119.4 (3)	C23—N2—H2A	106.0
C5—C6—H6	120.3	Cu1—N2—H2A	106.0
C7—C6—H6	120.3	N2—C23—C24	108.1 (2)
C8—C7—C6	120.9 (3)	N2—C23—H23A	110.1
C8—C7—H7	119.6	C24—C23—H23A	110.1
C6—C7—H7	119.6	N2—C23—H23B	110.1
C7—C8—C9	121.3 (3)	C24—C23—H23B	110.1
C7—C8—H8	119.3	H23A—C23—H23B	108.4
C9—C8—H8	119.3	N1—C21—C22	112.1 (2)
C8—C9—C4	117.9 (2)	N1—C21—H21A	109.2
C8—C9—C10	122.3 (2)	C22—C21—H21A	109.2
C4—C9—C10	119.8 (2)	N1—C21—H21B	109.2
C1—C10—C9	118.9 (2)	C22—C21—H21B	109.2
C1—C10—C11	119.4 (2)	H21A—C21—H21B	107.9
C9—C10—C11	121.7 (2)	O2—C24—C23	106.1 (2)
C20—C11—C12	119.6 (2)	O2—C24—H24A	110.5
C20—C11—C10	119.7 (2)	C23—C24—H24A	110.5
C12—C11—C10	120.7 (2)	O2—C24—H24B	110.5
C13—C12—C17	117.7 (2)	C23—C24—H24B	110.5
C13—C12—C11	123.2 (2)	H24A—C24—H24B	108.7
C17—C12—C11	119.1 (2)	O1—C22—C21	112.1 (2)
C14—C13—C12	121.2 (3)	O1—C22—H22A	109.2
C14—C13—H13	119.4	C21—C22—H22A	109.2
C12—C13—H13	119.4	O1—C22—H22B	109.2
C13—C14—C15	120.8 (3)	C21—C22—H22B	109.2
C13—C14—H14	119.6	H22A—C22—H22B	107.9
C15—C14—H14	119.6	C22—O1—H1A	109.5
C16—C15—C14	119.5 (3)	C24—O2—Cu1	118.73 (18)
C16—C15—H15	120.2	C24—O2—H2B	109.5
C14—C15—H15	120.2	Cu1—O2—H2B	126.1
			
C10—C1—C2—C3	−0.9 (4)	C12—C17—C18—C19	2.1 (4)
N1—C1—C2—C3	−177.5 (3)	C17—C18—C19—C20	−0.7 (4)
C1—C2—C3—C4	1.4 (5)	C12—C11—C20—C19	1.8 (4)
C2—C3—C4—C5	179.2 (3)	C10—C11—C20—C19	−175.5 (2)
C2—C3—C4—C9	0.0 (4)	C12—C11—C20—N2	179.8 (2)
C3—C4—C5—C6	−178.4 (3)	C10—C11—C20—N2	2.6 (4)
C9—C4—C5—C6	0.8 (4)	C18—C19—C20—C11	−1.3 (4)
C4—C5—C6—C7	0.7 (5)	C18—C19—C20—N2	−179.3 (2)
C5—C6—C7—C8	−1.2 (5)	C10—C1—N1—C21	141.6 (3)
C6—C7—C8—C9	0.2 (5)	C2—C1—N1—C21	−41.7 (3)
C7—C8—C9—C4	1.3 (4)	C10—C1—N1—Cu1	−84.7 (2)
C7—C8—C9—C10	−179.4 (3)	C2—C1—N1—Cu1	92.0 (3)
C3—C4—C9—C8	177.4 (3)	O2—Cu1—N1—C1	6.6 (4)
C5—C4—C9—C8	−1.8 (4)	N2—Cu1—N1—C1	59.88 (16)
C3—C4—C9—C10	−1.8 (4)	Cl2—Cu1—N1—C1	−101.97 (15)
C5—C4—C9—C10	179.0 (2)	Cl1—Cu1—N1—C1	148.49 (15)
C2—C1—C10—C9	−1.0 (4)	O2—Cu1—N1—C21	137.0 (3)
N1—C1—C10—C9	175.7 (2)	N2—Cu1—N1—C21	−169.67 (19)
C2—C1—C10—C11	179.9 (2)	Cl2—Cu1—N1—C21	28.49 (19)
N1—C1—C10—C11	−3.5 (4)	Cl1—Cu1—N1—C21	−81.05 (19)
C8—C9—C10—C1	−176.9 (2)	C11—C20—N2—C23	163.4 (2)
C4—C9—C10—C1	2.3 (4)	C19—C20—N2—C23	−18.6 (3)
C8—C9—C10—C11	2.2 (4)	C11—C20—N2—Cu1	−72.4 (3)
C4—C9—C10—C11	−178.5 (2)	C19—C20—N2—Cu1	105.6 (2)
C1—C10—C11—C20	66.2 (3)	O2—Cu1—N2—C20	−160.18 (19)
C9—C10—C11—C20	−112.9 (3)	N1—Cu1—N2—C20	31.60 (18)
C1—C10—C11—C12	−111.0 (3)	Cl2—Cu1—N2—C20	144.81 (17)
C9—C10—C11—C12	69.9 (3)	Cl1—Cu1—N2—C20	−62.04 (17)
C20—C11—C12—C13	−180.0 (3)	O2—Cu1—N2—C23	−30.02 (17)
C10—C11—C12—C13	−2.8 (4)	N1—Cu1—N2—C23	161.76 (17)
C20—C11—C12—C17	−0.4 (4)	Cl2—Cu1—N2—C23	−85.0 (2)
C10—C11—C12—C17	176.8 (2)	Cl1—Cu1—N2—C23	68.12 (16)
C17—C12—C13—C14	0.1 (4)	C20—N2—C23—C24	−178.8 (2)
C11—C12—C13—C14	179.7 (3)	Cu1—N2—C23—C24	50.4 (2)
C12—C13—C14—C15	0.1 (5)	C1—N1—C21—C22	−174.3 (2)
C13—C14—C15—C16	−0.1 (5)	Cu1—N1—C21—C22	59.3 (3)
C14—C15—C16—C17	0.0 (4)	N2—C23—C24—O2	−47.2 (3)
C15—C16—C17—C18	−178.1 (3)	N1—C21—C22—O1	55.6 (3)
C15—C16—C17—C12	0.2 (4)	C23—C24—O2—Cu1	21.4 (3)
C13—C12—C17—C18	178.1 (3)	N1—Cu1—O2—C24	59.7 (5)
C11—C12—C17—C18	−1.5 (4)	N2—Cu1—O2—C24	5.2 (2)
C13—C12—C17—C16	−0.3 (4)	Cl2—Cu1—O2—C24	169.1 (2)
C11—C12—C17—C16	−179.9 (2)	Cl1—Cu1—O2—C24	−81.9 (2)
C16—C17—C18—C19	−179.6 (3)		

### Hydrogen-bond geometry (Å, °)

**Table:**

*D*—H···*A*	*D*—H	H···*A*	*D*···*A*	*D*—H···*A*
O1—H1A···Cl1	0.84	2.41	3.039 (2)	132
O2—H2B···Cl1^i^	0.84	2.33	2.996 (2)	137
N2—H2A···O1^ii^	0.93	2.00	2.889 (3)	158

Symmetry codes: (i) −*x*+1, −*y*+1, −*z*+1; (ii) *x*−1, *y*, *z*.
